# Electronic Structure of Silicon Nanowires Matrix from Ab Initio Calculations

**DOI:** 10.1186/s11671-016-1238-7

**Published:** 2016-01-15

**Authors:** Liubomyr S. Monastyrskii, Yaroslav V. Boyko, Bogdan S. Sokolovskii, Vasylyna Ya. Potashnyk

**Affiliations:** Department of Radioelectronic and Computer Systems, Ivan Franko National University of Lviv, 50 Dragomanov Street, 79005 Lviv, Ukraine

**Keywords:** Porous silicon, Silicon nanowires, Electronic energy structure, Density functional theory, 81.05.Rm, 71.15-m, 73.21.Hb

## Abstract

An investigation of the model of porous silicon in the form of periodic set of silicon nanowires has been carried out. The electronic energy structure was studied using a first-principle band method—the method of pseudopotentials (ultrasoft potentials in the basis of plane waves) and linearized mode of the method of combined pseudopotentials. Due to the use of hybrid exchange-correlation potentials (B3LYP), the quantitative agreement of the calculated value of band gap in the bulk material with experimental data is achieved. The obtained results show that passivation of dangling bonds with hydrogen atoms leads to substantial transformation of electronic energy structure. At complete passivation of the dangling silicon bonds by hydrogen atoms, the band gap value takes the magnitude which substantially exceeds that for bulk silicon. The incomplete passivation gives rise to opposite effect when the band gap value decreases down the semimetallic range.

## Background

Quantum-dimensional structures based on Si, particularly Si nanowires, are attracting attention due to the possibility of creating on their basis of new optoelectronic devices. Optical properties of such devices are related to the size (diameter) of nanowires, distance between them that determines the degree of porosity, and behavior of foreign atom in these systems.

A feature of silicon nanowires is large surface area; therefore, the influence of surface conditions (defects, dangling bonds) is essential for the formation of the electronic structure and physical properties of systems based on them. Previous experimental and theoretical studies show the relationship between the size, structure, surface state, and band parameters of silicon nanowires [1].

One of the most powerful theoretical approaches to studying nanocrystals is the self-consistent calculations on the basis of the Kohn and Sham theory. For performing such calculations, a number of software packages using distributed multiprocessor computing powered by MPI and CUDA has been developed.

Nanoporous silicon with high degree of porosity can be satisfactorily described as a set of periodic array of silicon nanowires. In our work, we explored porous silicon taken in the form of such an array for two cases corresponding to the different degree of saturating silicon dangling bonds with hydrogen atoms.

## Methods

To determine the electronic structure of the objects under studying, we solved the self-consistent system of the Kohn-Sham equations [2]:

1 $$ h{\psi}_i(r)=\left(-\frac{1}{2m}{\nabla}^2+{V}_{eff}\left(r;n\right)\right){\psi}_i(r)={\varepsilon}_i{\psi}_i(r), $$

where { *ψ*_*i*_} are the orthonormal eigenfunctions, { *ε*_*i*_} are the corresponding eigenvalues of the single-particle Hamiltonian, *V*_eff_ is the effective potential expressed as a sum of the ionic potential *V*_ion_, the Hartree potential *V*_h_, and the exchange potential *V*_xc_:

2 $$ {V}_{\mathrm{eff}}\left(r;n\right)={V}_{\mathrm{ion}}\left(r;n\right)+{V}_{\mathrm{h}}\left(r;n\right)+{V}_{\mathrm{xc}}\left(r;n\right), $$

3 $$ n(r)=\sum_{i=1}^N\left|{\psi}_i{(r)}^2\right|. $$

The sum in (3) is applied to the lower *N* eigenvalues.

The corresponding secular equation (1) has the form

4 $$ \left(-\frac{1}{2m}{\nabla}^2+{V}_{\mathrm{ion}}+{V}_{\mathrm{h}}+{V}_{\mathrm{xc}}\right){\psi_{\mathrm{k}}}_n={\varepsilon}_{{{}_{\mathrm{k}}}_n}{\psi_{\mathrm{k}}}_n, $$

in which *V*_ion_ is the pseudopotential term

5 $$ {V}_{\mathrm{ion}}\left(r,r\hbox{'}\right)={\displaystyle \sum_{j,s}{V}_{\mathrm{ion}}^{(s)}}\left(r-{R}_j-{\tau}_s,r\hbox{'}-{R}_j-{\tau}_s\right), $$

where ${V}_{\mathrm{ion}}^{(s)}$ belongs to the *s*-th ion in the unit cell site, and *Rj*, *k*, and *n* denote the wave vector and band indices.

The local part of the Hartwigsen-Goedecker-Hutter pseudopotential [3] used in the calculations has the following form

6 $$ \begin{array}{l}{V}_{\mathrm{loc}}=-\frac{Z_{\mathrm{ion}}}{r}\mathrm{e}\mathrm{r}\mathrm{f}\left(\frac{r}{\sqrt{2}{r}_{\mathrm{loc}}}\right)+ \exp \left[-\frac{1}{2}{\left(\frac{r}{r_{\mathrm{loc}}}\right)}^2\right]\times \\ {}\times \left[{C}_1+{C}_2{\left(\frac{r}{r_{\mathrm{loc}}}\right)}^2+{C}_3{\left(\frac{r}{r_{\mathrm{loc}}}\right)}^4+{C}_4{\left(\frac{r}{r_{\mathrm{loc}}}\right)}^6\right],\end{array} $$

where erf denotes the error function, *C*_*i*_ are the parameters of the potential. The nonlocal contribution to the pseudopotential is described by the expression

7 $$ {V}_l\left(r,r\hbox{'}\right)={\displaystyle \sum_{i=1}^3{\displaystyle \sum_{j=1}^3{\displaystyle \sum_{m=-l}^{+l}{Y}_{l,m}(r){p}_i^l(r){h}_{i,j}^l}}}{p}_i^l\left(r\hbox{'}\right){Y}_{l,m}^{*}\left(r\hbox{'}\right). $$

In (7), *Y*_*l*,*m*_ are the spherical harmonics and ${p}_i^l(r)$ are the Gaussians in the following form

8 $$ {p}_i^l(r)=\frac{\sqrt{2}{r}^{l+2\left(i-1\right)} \exp \left(\frac{-{r}^2}{2{r}_l^2}\right)}{r_l^{l+\left(4i-1\right)/2}\sqrt{\varGamma \left(l+\frac{4i-1}{2}\right)}}, $$

where *Γ* is the gamma function.

Optimization of the structure was carried out by the BFGS method (Broyden-Fletcher-Goldfarb-Shanno) [4–7] according to which current search of the direction *p*_k_ is determined by solving the equation

9 $$ {B}_k{p}_k=-\nabla f\left({x}_k\right), $$

where *B*_*k*_ is the approximate Hessian matrix calculated at each iteration step.

Convergence of the self-consistent cycle of calculations was determined by the difference in full energies obtained in two successive stages which was chosen less than 10^−6^ eV.

We used for calculation the hybrid exchange-correlation potential (B3LYP). This form of exchange-correlation potential allows us to reach more accurate results as compared to those obtained at using other approximations. Modeling was performed for the case of ideal lattice of Si nanowires with transverse dimension 5 × 5 (the size is indicated in the units of cell parameter which is equal to 5.43 Å). The distance between nanowires was chosen sufficiently large in comparison with the nanowire diameter for ensuring the absence of interaction between the nanowires.

## Results and Discussion

In our calculations, we have assumed the following type of nanocrystalline interface: dangling bonds at the border of nanowire are saturated with hydrogen and the nanocrystals are in vacuum. This leads to formation of electronic confinement when a carrier does not able to withdraw the nanocrystal.

The distance between the quantum wires was comparable with lateral dimension of the wires. The surface was passivated with hydrogen that allowed to avoid the appearance of surface states in the band gap.

**Fig. 1:**
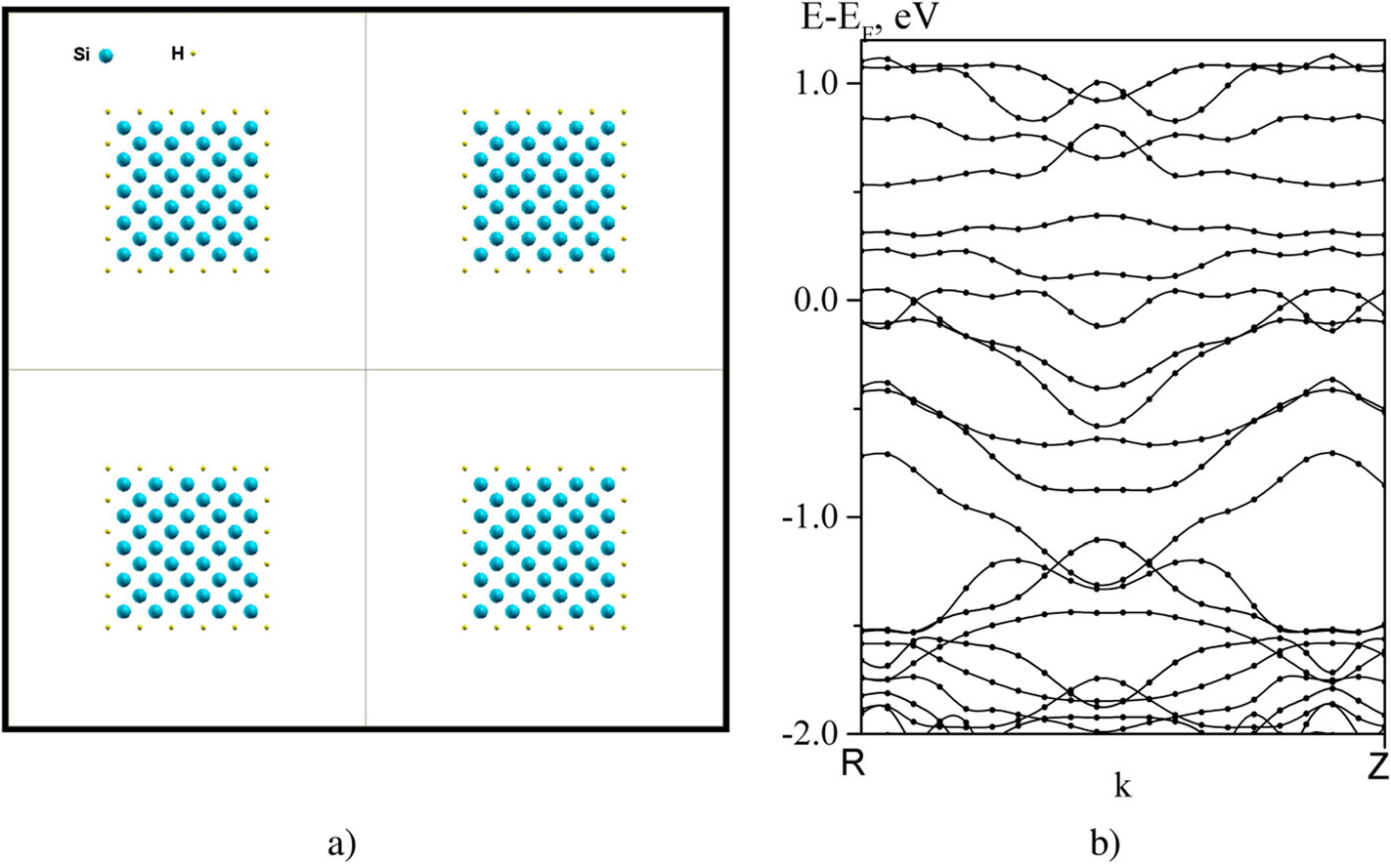
**a** Schematic of array of nanowires with orientation [100] and low degree of saturation with hydrogen. **b** Band structure of the array

**Fig. 2:**
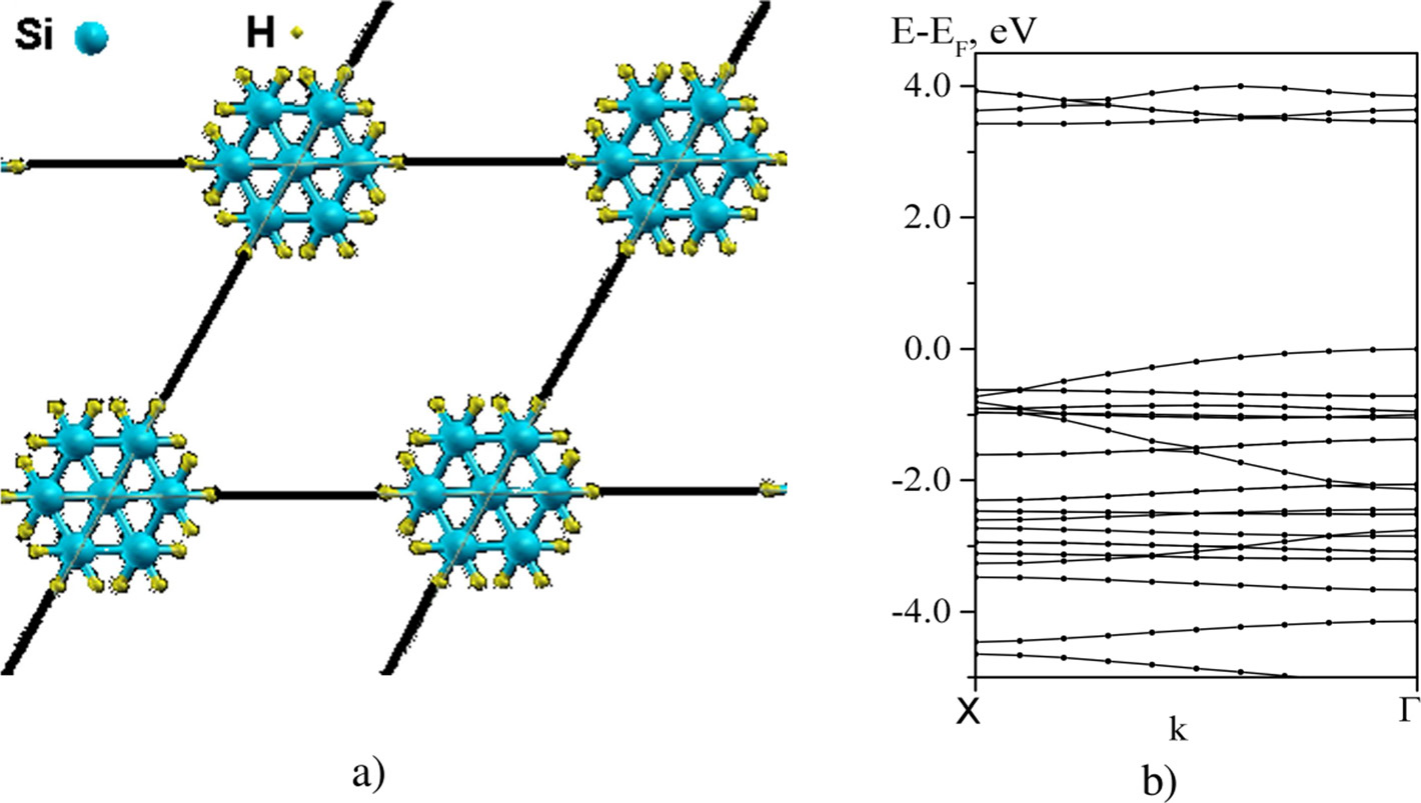
**a** Schematic of array of nanowires with orientation [111]. **b** Band structure of the array

The results of calculations are presented in Figs. 1 and 2. Fig. 1a shows the top view of the matrix of PS nanowires with a period of about 0.5 nm which are oriented along [111] direction. The high degree of passivation of the dangling silicon bonds (*N*_*Н*_*/N*_*Si*_) was observed. The content of nanowires at the surface corresponded to the Si_7_H_18_ composition, and the porosity was equal to 87.5 %. The calculated electron energy structure of this matrix is presented in Fig.1b. The energy band gap is seen to amount to approximately 3.5 eV that substantially exceeds the energy band gap of bulk silicon (~1.1 eV at room temperature).

In the case of low degree of passivation of the dangling silicon bonds by hydrogen *N*_*Н*_*/N*_*Si*_ ~ 50 at [100] orientation (Fig. 2a) when the surface content corresponded to the Si_41_H_20_ composition and porosity was 75 %, the energy band structure has a quasisemimetallic character, i.e., band gap approaches to zero (Fig. 2b). In this case, the cardinal change of energy spectrum of PS nanowire matrix for different degrees of passivation of surface atoms by hydrogen is realized. Actually, such an effect can be observed at the initial stages of formation of PS. Then, the aging process takes place, i.e., oxidation of the surface of PS and stabilization of its electron structure.

Quasisemimetallic nature of the band structure can be explained by restrictions of the density functional theory, since the used approach gives the correct value of the band gap for bulk silicon. Similar to the results of [8], it can be assumed that the main contribution to the formation of such a form of the band structure is due to the unsaturated bonds of the surface atoms of silicon.

Large value of the energy gap is related to the quantum restriction (quantum confinement) of charge carriers. The presence of the direct band gap can be explained by the differences in the anisotropy of band structures of bulk silicon and silicon nanowires. In bulk silicon, the longitudinal effective mass in the conduction band by a factor of 4 exceeds the transverse effective mass, whereas in silicon nanowires, the transverse minimum of conduction band has larger effective mass than that for the longitudinal minimum. The band with more effective mass shows less energy shift due to the quantum restriction [9]. These features of the band structure of silicon quantum silicon nanowires can be a basis for creation of direct band semiconductor materials.

The effects of substantial decrease of the energy band gap were observed in [10] for the Si nanocrystals containing additional lithium atoms. The most strong effect was observed at the incorporation of lithium at the expense of the singlet level that strongly shifted down which gives rise to decreasing the optical band gap.

In our case of incomplete passivation, overlapping of the levels of valence and conduction bands of Si nanowires takes place.

## Conclusions

In this paper, self-consistent calculation of electronic energy structure of quantum-dimensional periodic matrix of silicon nanowires has been carried out with using the first principles. The obtained results show that passivation of dangling bonds with hydrogen atoms leads to substantial transformation of electronic energy structure. At complete passivation of the dangling silicon bonds by hydrogen atoms, the band gap value takes the magnitude which substantially exceeds that for bulk silicon. The incomplete passivation gives rise to opposite effect when the band gap value decreases down the semimetallic range.
